# Response to the Letter to the Editor by Dunning Hotopp and Klasson

**DOI:** 10.1534/g3.117.300379

**Published:** 2018-01-03

**Authors:** Wilson Leung, Sarah C. R. Elgin

**Affiliations:** Department of Biology, Washington University in St. Louis, Missouri 63130

In Leung *et al.* (2017), we provide evidence that *Drosophila* transposons have been major contributors to the expansion of the *Drosophila ananassae* fourth chromosome (Muller F element) arms. Dunning Hotopp and Klasson agree with this finding, but they take exception to our second conclusion, that lateral gene transfer (LGT) from the *Wolbachia* endosymbiont of *D. ananassae* (*wAna*) had only a minor role in the expansion of the *D. ananassae* fourth chromosome arms. This disagreement might have arisen because of a misunderstanding of the scope of our report. As stated in the paper, our analysis is restricted to the “assembled portions” of the *D. ananassae* F element (∼18.7 Mb, all within the chromosome arms, not including the telomeric and pericentromeric regions). We did not generalize our findings to the whole F element. Hence, we agree with Dunning Hotopp and Klasson that the results reported in Leung *et al.* (2017) do not preclude the possibility that LGT from *wAna* is a contributor to the expansion of the *D. ananassae* F element in its entirety; specifically, LGT could be contributing to an expansion of the pericentric heterochromatin regions of the F element, as reported in Klasson *et al.* (2014).

Because we only manually improved ∼1.4 Mb of the *D. ananassae* F element, *Wolbachia* sequences that are integrated into the *D. ananassae* F element could be located within gaps or unassembled portions. However, the presence of large-scale LGT from *wAna* in the manually improved regions of the *D. ananassae* F element is very unlikely, as the gap sizes in the improved regions were estimated by multiple restriction digests, or by long reads produced by the Pacific Biosciences (PacBio) sequencer.

We did find several scaffolds within the *D. ananassae* CAF1 assembly that show sequence similarity to the *Wolbachia* endosymbiont of *D. melanogaster* (*wMel*) along the entire length of the scaffold (*e.g.*, scaffold_12940). Hence, if sequencing reads that exhibit sequence similarity to *wMel* were filtered as part of the sequencing or assembly pipeline, as suggested by Dunning Hotopp and Klasson, then the parameters that were used allow at least some of the *wMel* reads to be assembled in the CAF1 assembly. We note also that most of the telomeric and pericentric heterochromatin regions are missing from the CAF1 assembly. Thus, our findings do not contradict the work by Klasson and colleagues, who used fluorescence *in situ* hybridization of *Wolbachia* to conclude that “[h]ybridization to the fourth chromosome is consistent with the LGT being largely heterochromatic (Figure 9)” (Klasson *et al.* 2014), describing hybridization to the pericentric region. Both sources of expansion could well have occurred in different regions of the F element, with retrotransposons contributing to the expansion of the chromosome arms and LGT from *wAna* contributing to an expansion of the heterochromatic regions.

We certainly agree with Dunning Hotopp and Klasson that higher quality genome assemblies for *D. ananassae* and for *wAna* are needed in order to ascertain the extent to which LGT from *Wolbachia* contributes to the expansion of the *D. ananassae* F element overall. For example, assemblies based on long reads produced by PacBio or Nanopore sequencers might enable the clear identification of *Wolbachia* sequences that are integrated into the *D. ananassae* genome. Nonetheless, even with long-read technologies, it will remain difficult to generate a high-quality assembly for the *D. ananassae* pericentric heterochromatin region and, as such, to get a reliable estimate of the level of LGT from *Wolbachia* in these regions. However, within the assembled portions of the chromosome arms, which contain 64 complete *D. ananassae* F element genes, the available data argue that retrotransposon expansion was the major driver of chromosome expansion.

*Note added in proof:* See Dunning Hotopp and Klasson in this issue for a related work.
